# Early life overfeeding impairs spatial memory performance by reducing microglial sensitivity to learning

**DOI:** 10.1186/s12974-016-0578-7

**Published:** 2016-05-18

**Authors:** Simone N. De Luca, Ilvana Ziko, Luba Sominsky, Jason C. D. Nguyen, Tara Dinan, Alyson A. Miller, Trisha A. Jenkins, Sarah J. Spencer

**Affiliations:** School of Health and Biomedical Sciences, RMIT University, Melbourne, Vic. 3083 Australia

**Keywords:** Microglia, Radial arm maze, Obesity, Neonatal overfeeding, Neurogenesis, Inflammation

## Abstract

**Background:**

Obesity can lead to cognitive dysfunction including poor performance in memory tasks. However, poor memory is not seen in all obese humans and takes several months to develop in animal models, indicating the adult brain is relatively resistant to obesity’s cognitive effects. We have seen that, in the rat, overfeeding for as little as 3 weeks in early life leads to lasting obesity and microglial priming in the hypothalamus. Here we hypothesized that microglial hyper-sensitivity in the neonatally overfed rats extends beyond the hypothalamus into memory-associated brain regions, resulting in cognitive deficits.

**Methods:**

We tested this idea by manipulating Wistar rat litter sizes to suckle pups in litters of 4 (overfed) or 12 (control).

**Results:**

Neonatally overfed rats had microgliosis in the hippocampus after only 14 days overfeeding, and this persisted into adulthood. These changes were coupled with poor performance in radial arm maze and novel object recognition tests relative to controls. In controls, the experience of the radial arm maze reduced cell proliferation in the dentate gyrus and neuron numbers in the CA3. The learning task also suppressed microglial number and density in hippocampus and retrosplenial cortex. Neonatally overfed brains had impaired sensitivity to learning, with no neuronal or cell proliferative effects and less effective microglial suppression.

**Conclusions:**

Thus, early life overfeeding contributes to a long-term impairment in learning and memory with a likely role for microglia. These data may partially explain why some obese individuals display cognitive dysfunction and some do not, i.e. the early life dietary environment is likely to have a vital long-term contribution.

**Electronic supplementary material:**

The online version of this article (doi:10.1186/s12974-016-0578-7) contains supplementary material, which is available to authorized users.

## Background

Obesity, now epidemic in our population, is associated with deficits in cognitive processing, including in learning, memory, and executive function [1–5]. It has also been linked to a greater risk of dementia and Alzheimer’s disease later in life [6–8]. However, not all obese subjects develop cognitive deficits [9, 10], and factors that make individuals vulnerable to this complication of obesity are largely unknown. We suggest that early life diet may be important in programming cognitive function with obesity.

Recent studies have suggested inflammation may contribute to cognitive dysfunction in some obese subjects [11]. Systemic markers of chronic low-grade inflammation have been linked with poor cognitive ability in obese humans [12, 13]. In rodent models, microgliosis (microglial proliferation, accumulation, and activation [14, 15]), astrogliosis, and elevated tumour necrosis factor (TNF)α protein are seen in the hippocampi of mice fed a high-fat diet long term [16, 17]. These mice also have impaired spatial memory function in behavioural tests, with high-fat diet-fed mice taking longer to learn the location of an escape platform in the Morris Water Maze and recalling their training poorly in the probe trial [16, 18]. Importantly for the idea that the inflammatory and behavioural effects of high-fat diet are linked, these cognitive deficits, as well as the microgliosis and elevated cytokines, are significantly improved by treatment with anti-oxidant anti-inflammatory agents such as resveratrol or ursolic acid [16, 18].

Brain and behavioural studies thus suggest a link between obesity/high-fat diet, central inflammation, and cognition, but there is also evidence of cognitive resilience under these conditions. Cognitive dysfunction is not seen in all studies of obese humans [9, 10], and in adult rodents a short-term high-fat diet has little to no effect on extra-hypothalamic inflammation, with microglial, astrocyte, and cytokine markers being elevated in the hypothalamus, but not other brain regions examined, including cortex and hippocampus, after 2 or 3 weeks high fat [19]. In the studies mentioned above where diet was associated with hippocampal inflammation and behavioural deficits partially reversible with anti-inflammatories, the rats consumed a high-fat diet for 20 and 22 weeks prior to testing [16, 18], suggesting hippocampal inflammation and cognitive dysfunction take much longer to develop with high-fat intake than hypothalamic deficits.

We have previously shown that the early life period is one of the particular vulnerability to the long-term effects of diet. Early life overfeeding and the obesity that ensues results in pronounced hypothalamic inflammation, with microgliosis in the paraventricular nucleus of the hypothalamus (PVN) and an increase in the hypothalamic expression of pro-inflammatory genes [20, 21]. Although evidence suggests that the effect of an adult high-fat diet is somewhat reversible [22, 23], at least in the hippocampus, we have shown that rats that are overfed as neonates retain hypothalamic microglial priming into adulthood despite the resumption of a normal diet at weaning. They also have a significantly more reactive central and peripheral immune response to inflammatory challenge throughout life [20, 21, 24].

Given that we see such pronounced and lasting inflammation in the hypothalamus after neonatal overfeeding [20, 21], we hypothesized that early life diet would also be important for programming long-term hippocampal inflammation and that this might lead to cognitive deficits. In this investigation, we therefore examined cognitive function in the novel object recognition, delayed win-shift radial arm maze (RAM), and contextual fear conditioning tests in adult rats made obese due to neonatal overfeeding. We then examined the effects of one of these learning tests (the RAM) on neuronal proliferation and microglial profiles to assess how neonatal overfeeding impacts on the ability to modulate learning-associated brain regions, hippocampus, retrosplenial cortex, and amygdala [25, 26] to facilitate memory.

## Methods

### Animals

We obtained timed pregnant Wistar rats from the Animal Resources Centre, WA, Australia. On arrival at the RMIT University Animal Facility, they were housed at 22 °C on a 12 h light/dark cycle (0700–1900 h) and provided with ad libitum pelleted rat chow and water. We conducted all procedures in accordance with the National Health and Medical Research Council Australia Code of Practice for the Care of Experimental Animals and the RMIT University Animal Ethics Committee approval.

### Litter size manipulation

On the day of birth (postnatal day (P)0), we removed all pups from their dams and randomly reallocated them to new dams in litters of 12 (control litter (CL)) or 4 (small litter (SL), neonatal overfeeding) as we have previously described [27–29]. Dams did not receive any of their own pups, and each new litter was made up of 50 % males and 50 % females. Excess pups were culled. We have shown that this manipulation results in SL pups being significantly heavier by P7 and heavier throughout life [27–29]. We have previously reported weight data from this cohort of rats and have analysed their hypothalami for indices of microgliosis [20].

The pups were either used for experimentation at P7 or 14 or allowed to grow into adulthood, approximately P70. In this latter case, we separated the pups into same-sex littermate pairs upon weaning at P21 and left them undisturbed until experimentation, except for the usual animal husbandry. We used only males in these experiments, keeping the females for use in other studies. We derived all experimental groups from three or more litters, using a maximum of two pups from the same litter for an experimental treatment.

### Effects of neonatal overfeeding on neonatal and adult hippocampal microgliosis, neurogenesis, and central inflammatory gene expression

On P7, P14, or approximately P70 (including after conclusion of the RAM testing, see below), we deeply anesthetized a cohort of rats with Lethabarb (150 mg/kg pentobarbitone sodium, i.p.). For immunohistochemical analysis of changes in microglia and neurons, we perfused these rats transcardially with phosphate-buffered saline (PBS 4 °C, pH 7.4), followed by 4 % paraformaldehyde in PBS, (PBS 4 °C, pH 7.4). Brains were removed and post-fixed for 24 h in the same fixative before placing them in 20 % sucrose in PBS (4 °C). We then cut the forebrains into 40 μm (neonates) or 30 μm (adults) coronal sections using a cryostat. Sections were cut in a one in five series and were stored at 4 °C until use. *N* = 6–12 per group.

We also examined changes in hippocampal gene expression after neonatal overfeeding at these ages. We deeply anaesthetized a second cohort of pups, dissected hippocampi on ice and immediately snap-froze them for real-time reverse transcriptase polymerase chain reaction (qrt-PCR). The brains used for rt-PCR were not perfused; however, the dissection was conducted in PBS over ice to remove excess blood. All experiments took place between 0900 and 1300 h to limit potential effects of circadian rhythms on any parameters measured. *N* = 6–8 per group.

### Immunohistochemistry

Sections through the hippocampus were immunolabelled for ionized calcium-binding adapter molecule-1 (Iba-1), a marker for microglia/macrophages; neuronal nuclei (NeuN), a marker for total neuron numbers; doublecortin (DCX), a marker for developing neurons; Ki67, a cell proliferation marker; activated caspase-3, a marker of apoptosis; or synaptophysin, a pre-synaptic terminal marker. Iba-1 is a commonly used marker for identification of microglia [30–32]. It is clearly constitutively expressed in microglia and is not expressed in neurons, astrocytes, or oligodendroglia [33, 34]. However, we should note that it is expressed on cells of the monocyte/macrophage lineage, including non-microglial CNS macrophages. Randomly selected sections from each treatment group were processed at the same time in batches. Briefly, we incubated a series of sections 150–200 μm apart from each animal in primary antibody overnight (Iba-1: 1:1000, rabbit, 4 °C, Wako Chemicals USA, Inc., Richmond, VA, USA; NeuN: 1:5000, rabbit, 4 °C, Abcam, Cambridge, England, UK; DCX: 1:500, goat, 4 °C, Santa Cruz Biotechnology Inc., Dallas, TA, USA; Ki67: 1:500, rabbit, room temperature (RT), Abcam; Activated caspase-3: 1:250, rabbit, RT, Abcam; Synaptophysin: 1:1000, mouse, RT, Sigma-Aldrich, St Louis, MO, USA). This was followed by secondary antibody (Iba-1, NeuN: 1.5 h, 1:200, biotinylated anti-rabbit, Vector Laboratories, Burlingame, CA, USA; DCX: 1.5 h, 1:500, biotinylated anti-goat, Vector; Ki67, caspase-3: 2 h, 1:500, Alexa Fluoro 594 anti-rabbit, Life Technologies, Burlingame, CA, USA; Synaptophysin: 2 h 1:500, Alexa Fluoro 488 anti-mouse, Life Technologies). For Iba-1, NeuN, and DCX, we used an avidin-biotin horseradish peroxidase (HRP) complex (ABC; 45 min; Vector Elite kit; Vector) followed by diaminobenzidine (DAB) to visualize the HRP activity, seen as amber staining. We stopped the reaction when the contrast between specific cellular and non-specific background labelling was optimal. We mounted the sections on polylysine-coated slides, air-dried them, dehydrated them in a series of alcohols, cleared them in histolene, and coverslipped. For Ki67, caspase-3, and synaptophysin, we used DAPI as a nuclear counterstain (10 min; 1:2000 from 5 mg/mL stock), followed by immediate coverslipping with Dako fluorescence mounting medium (Dako, Glostrup, Denmark).

Hippocampal and retrosplenial cortex sections were assessed by an experimenter blinded to treatment groups for numbers and density of cells positive for Iba-1 and for numbers of cells positive for NeuN, DCX, caspase-3, and Ki67. For Iba-1, we assessed these as we have previously described [20]. Briefly, we used the thresholding method on photomicrograph images imported into image analysis software ImageJ (National Institutes of Health, Bethesda, MD, USA). For each region, we selected a sub-region of interest (ROI), identified according to the Paxinos and Watson Rat Brain Atlas [35], and analysed six sections 120 μm apart between 2.76 and 3.48 mm caudal to bregma per animal. To exclude the possibility of brain or regional atrophy or swelling, we also assessed brain and hippocampal dimensions (width and height) for these sections. We found no differences between the groups (data not shown). We saw no differences between the rostrocaudal levels for any of the hippocampal regions, so we took the summed counts and mean density of the six images as our sampled result. For synaptophysin, we employed the thresholding method on ROIs through the hilus (three sections 120 μm apart between 2.76 and 3.48 mm caudal to bregma) to determine the intensity of immunofluorescence (NIS-Elements AR software; Nikon, USA). We also used the thresholding method for NeuN through the dentate gyrus.

### Gene expression

To determine changes in candidate genes involved in central inflammation, we isolated RNA from hippocampi using QIAzol and an RNeasy purification kit (QIAGEN, Valencia, CA, USA). RNA (1 μg) was transcribed to complementary DNA (cDNA) using an iScript cDNA synthesis kit (Bio-Rad Laboratories, Hercules, CA, USA), following the manufacturer’s instructions. rt-PCR was performed using Taqman Gene Expression Assays (Applied Biosystems, Mulgrave, Vic, Au). The specific primer details are shown in Table 1. Fold differences in target messenger RNA (mRNA) expression were measured using the delta-cycle threshold method by comparison with the housekeeping gene, *18S* [36, 37], and expressed as mRNA relative fold change as described previously [20, 38].

**Table 1:** TaqMan probe details (Life Technologies) used for qr-PCR

Target gene	NCBI reference sequence	TaqMan assay ID	Product size
*Bdnf*	NM_001270630.1	Rn02531967_s1	142
*Il10*	NM_012854.2	Rn01483988_g1	105
*Il1β*	NM_031512.2	Rn00580432_m1	74
*Il1rn (I1lra)*	NM_022194.2	Rn02586400_m1	77
*Il6*	NM_012589.2	Rn01410330_m1	121
*Nfkb1*	NM_001276711.1	Rn01399583_m1	63
*Tlr4*	NM_019178.1	Rn00569848_m1	127
*Tnfα*	NM_012675.3	Rn01525859_g1	92
*18 s*	X03205.1	4319413E	187

### Behaviour

Y maze: To assess if neonatal overfeeding influenced spatial memory, we tested the rats in a Y maze task, which exploits the rats’ natural tendency to explore novel environments. The Y maze was a three-arm maze with equal angles between all arms (50 cm long × 17 cm wide × 32 cm high) and spatial cues external to the maze. We habituated the rats to the maze twice on consecutive days for 5 min. On the test day (day 3), we allowed the rats to explore the maze for 10 min, having access to two of the three arms. They were then returned to their home cages for a 4 h inter-trial interval (ITI) during which the maze was cleaned with 70 % ethanol. The rats were then placed back into the maze for 5 min, this time having access to all arms. We filmed both trial and test phases for subsequent behavioural analysis. An experimenter blinded to treatment group assessed the number of entries into the novel arm and the time the rat spent in each arm. *N* = 10 per group.

Novel object recognition: To examine the effects on working memory, we next tested a separate cohort of rats in the novel object recognition task [39]. This test also exploits the rats’ attraction to novelty, in this case a novel object in the same context. We first gave the rats two sessions of 3 min habituation to an empty open field arena (a black plywood box, 65 cm × 65 cm × 65 cm) on the days preceding the test. On the testing day, we gave the rats a 3 min acquisition trial in the same arena with two identical objects. Objects were placed in the centre of the box equal distances from all sides. Following a 1 h ITI, we returned the rats to the arena with one familiar object and one novel object for a 3 min retention test. Between each phase and each rodent, the arena was cleaned with 70 % ethanol. All sessions were filmed, and an experimenter blinded to treatment group scored the videos for time spent interacting with each object. Results from the retention phase were expressed as a discrimination index calculated as time spent interacting with novel object minus time spent interacting with familiar object divided by the overall exploration time of the two objects in seconds. *N* = 10 per group.

Delayed win-shift RAM: To assess spatial reference and working memory, we also tested the rats in a delayed spatial win-shift procedure on the RAM, adapted from Packard et al. [40]. Testing was carried out in an eightarm radial maze, consisting of an octagonal central platform (34 cm diameter) and eight equally spaced radial arms (87 cm long, 10 cm wide). At the end of each arm was a food well (2 cm in diameter and 0.5 cm deep). At the start of each arm was a clear Perspex door that controlled access in and out of the central area. Each door was controlled by a computerized control system enabling the experimenter to regulate access to the arms. Salient visual cues of different geometric shapes and contrasting colours were placed around the maze on the walls of the room.

On the first day of testing, rodents were habituated to the maze in two 10 min sessions. After the final habituation session of the day, the rodents were returned to their home cages and given approximately 20 grain reward pellets (45 mg; Bio-Serv, Flemington, NJ, USA). Following habituation, rodents underwent two trials per day for 14 consecutive days consisting of a 5 min training phase, a 5 min ITI where the rat was returned to the home cage, and a 5 min test phase. Before the training phase, four arms were pseudo-randomly chosen and blocked, with no more than two adjacent arms blocked. The four unblocked arms were baited with grain reward pellets. For the training phase, we gave each rat 5 min to enter and retrieve the grain pellet rewards from all the baited arms. After a 5 min ITI, we reintroduced the rat to the maze, this time with all eight arms opened and the previously blocked arms baited with grain reward pellets. We recorded the number of arm entries for each rat in real time. An arm entry was recorded when the animal had moved all four paws off the central platform into the arm. Two types of errors were recorded: working error (re-entry of an arm that had been baited and visited) and reference error (entry into an arm that was baited during the training phase). We continued RAM testing until the CL rats made no more than one error in the test phase. *N* = 10 per group. Twenty-four hours after the last RAM trial (25 trials total), we perfused the rats, and a non-RAM control group, to assess learning-induced changes in neuron and microglia numbers as described above.

Contextual fear conditioning: We also tested a separate cohort of CL and SL rats in a contextual fear conditioning test for hippocampal and amygdala-dependent memory. We placed each rat individually in a conditioning chamber (Coulbourn Instruments, Lehigh Valley, PA, USA) for a 3 min baseline trial and then gave them three unsignalled footshocks of 2 s each with a 60 s inter-stimulus interval (0.5 mA shocks) through a metal grid floor. We recorded spontaneous motor activity (crosses through the midline of the chamber) and vertical exploration (rearing) during the conditioning trial. Every 8 s after each footshock, we scored each rat for defensive freezing, which was defined as cessation of all movement except that required for breathing. We returned the rats to their home cage 60 s after the last footshock and cleaned the conditioning apparatus with 70 % ethanol. Twenty-four hours later, we again placed the rats into the conditioning chamber for the extinction trial. They were scored for defensive freezing every 8 s for 8 min. Both post-shock and extinction trial scores were converted to a percentage time spent freezing in each of the trials. Extinction trial freezing scores are presented as a percentage of post-shock freezing, with scores <100 % representing less percentage time freezing during the extinction trial than in the post-shock, consistent with poor memory of the event [41]. *N* = 6–10 per group.

### Data analysis

We analysed neonatal and adult changes separately. For neonates, we compared Iba-1 cell counts and density for each region and gene expression data using two-way analyses of variance (ANOVA)s with neonatal nutritional environment (CL/SL) and age (P7/14) as between factors. Where significant interactions were found, we then performed Tukey’s post hoc tests. For the adults, we used Student’s unpaired *t* tests to assess CL/SL cell counts and density for each region, gene expression, Y maze, novel object recognition and contextual fear conditioning, and two-way ANOVAs to assess post-RAM changes with litter size and RAM as between factors and Tukey’s post hoc tests. For the RAM, training and test phase data were binned into blocks of four and data analysed using two-way ANOVAs with litter size and block as between factors, followed by Tukey’s post hoc tests as described [42]. We also performed Pearson’s correlational analyses for the RAM data to assess if there were any correlations between learning and weight, microglia counts or density in any region, Ki67, or DCX. Data are presented as the mean ± SEM. Statistical significance was assumed when *p* ≤ 0.05.

## Results

### Neonatal overfeeding leads to microgliosis in the CA1 hippocampus at P14

**Fig. 1:**
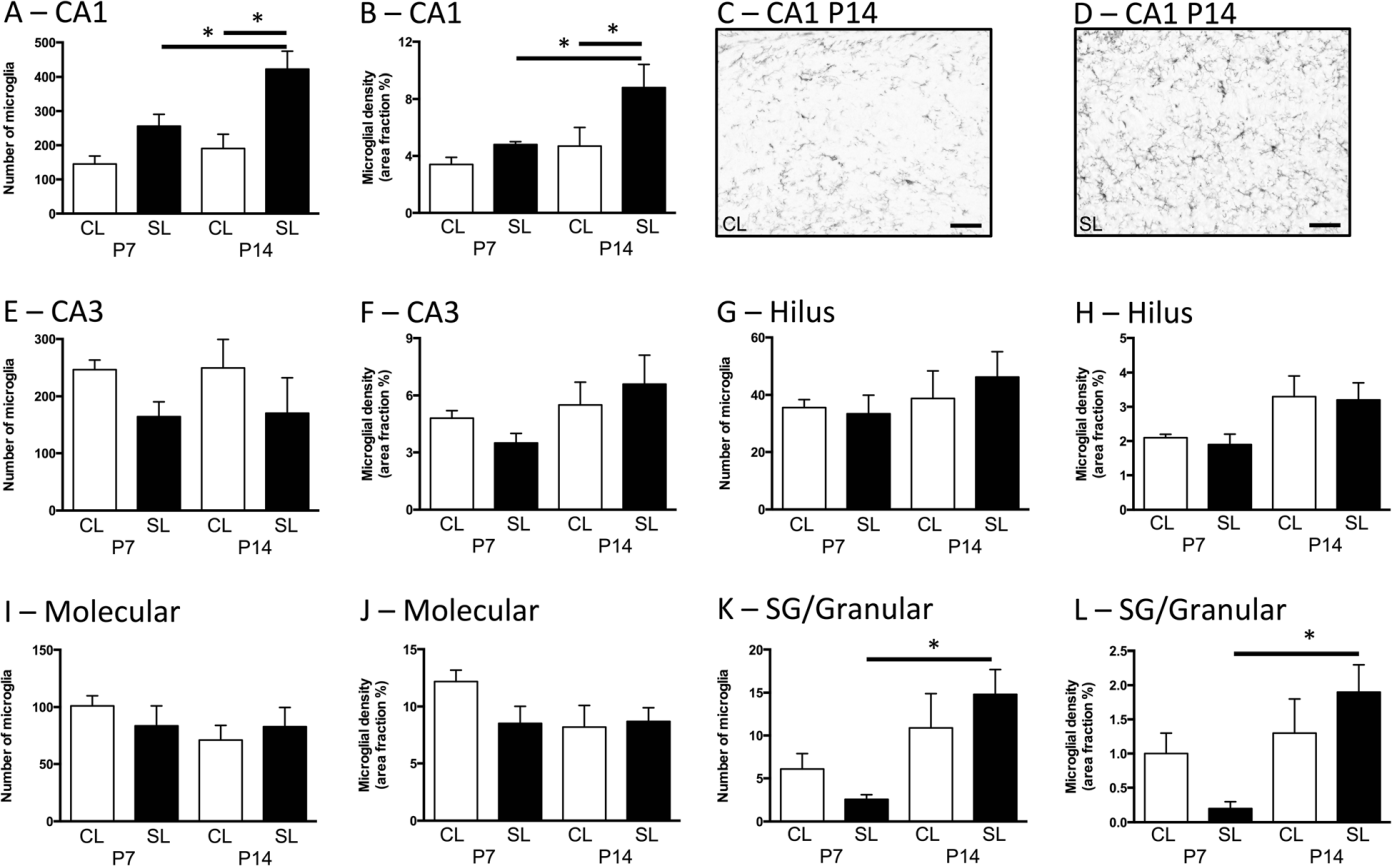
Numbers and density of ionized calcium-binding adapter molecule-1 (Iba-1)-stained cells at postnatal days (P)7 and 14 in rats raised in control (CL) and small (SL) litters. **a**–**d** CA1. **e**, **f** CA3. **g**, **h** Dentate gyrus (DG) hilus. **i**, **j** DG molecular region. **k, l** DG sub-granular (SG)/granular region. Data are mean + SEM. *N* = 6–12 per group. *p < 0.05. **c**, **d** Representative photomicrographs of the CA1 region from P14 rats illustrating differences in numbers and density of Iba-1-stained cells. *Scale bars* = 50 μm

To investigate the acute effects of neonatal overfeeding on central inflammation, we examined microglial profiles in several regions of the hippocampus in CL and SL rats. Neonatal overfeeding increased both microglial number and density in the CA1 at P14 compared with CL, and both were also increased in SL at P14 with respect to P7 (number: significant effect of litter size (*F*_(1,29)_ = 21.76, *p* < 0.001) and age (*F*_(1,29)_ = 8.31, *p* = 0.007); density: significant effect of litter size (*F*_(1,29)_ = 8.01, *p* = 0.008) and age (*F*_(1,29)_ = 7.67, *p* = 0.010); Fig. 1a–d). There were main effects of litter size and age in the other regions, but no specific effects of neonatal overfeeding with post hoc comparisons. Thus, in the CA3, neonatal overfeeding suppressed microglial numbers overall (*F*_(1,24)_ = 4.76, *p* = 0.039; Fig. 1e), and neonatal age led to an increase in density overall (*F*_(1,24)_ = 4.79, *p* = 0.039; Fig. 1f). Density also increased overall with age in the hilus of the dentate gyrus (DG; *F*_(1,24)_ = 13.07, *p* = 0.001; Fig. 1h), and microglial numbers (*F*_(1,24)_ = 13.18, *p* = 0.001; Fig. 1k) and density (*F*_(1,24)_ = 4.68, *p* = 0.041; Fig. 1l) were increased with age in the sub-granular/granular zone, with specifically more microglia and more dense microglia in the P14 SL sub-granular/granular region than in P7 SL.

### Effects of neonatal overfeeding on neonatal hippocampal pro-inflammatory gene expression

**Fig. 2:**
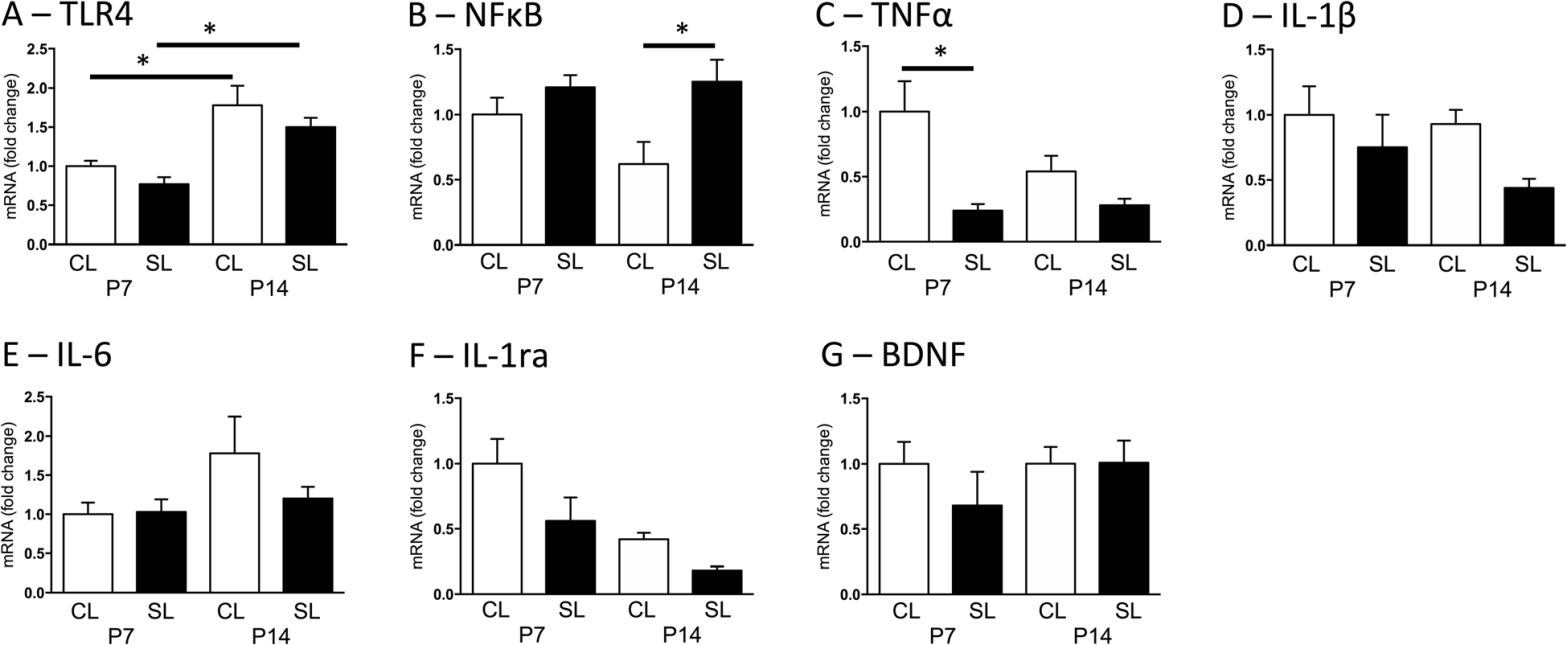
Hippocampal expression of **a** toll-like receptor 4 (*Tlr4*), **b** nuclear factor (*NF) κB*, **c** tumour necrosis factor (*TNF)α*, **d** interleukin (*IL)-1β*, **e** IL-6, **f** *IL-1ra*, and **g** brain-derived neurotrophic factor (*Bdnf*) at postnatal days (P)7 and 14 in rats raised in control (CL) and small (SL) litters. Data are mean + SEM. *N* = 6–8 per group. *p < 0.05

To investigate if these acute changes in CA1 microglia with neonatal overfeeding were associated with changes in hippocampal expression of pro-inflammatory genes, we examined expression of a suite of these. Toll-like receptor 4 (*Tlr4*) was elevated in the hippocampus in both CL and SL at P14 compared with P7 (litter size *F*_(1,28)_ = 4.31, *p* = 0.047 and age *F*_(1,28)_ = 32.47, *p* < 0.001 effects; Fig. 2a). Neonatal overfeeding led to a significant elevation of nuclear factor κB (*Nfκb*), an important regulator of pro-inflammatory gene transcription, at P14 compared with CL (litter size effect *F*_(1,26)_ = 9.98, *p* = 0.004; Fig. 2b) and a suppression of tumour necrosis factor α (*Tnfα*) at P7 compared with CL (litter size effect *F*_(1,26)_ = 7.21, *p* = 0.012; Fig. 2c). There were no notable differences between the groups in hippocampal interleukin (IL)-*Il1β*, *Il6*, *Il1rn*, or brain-derived neurotrophic factor (*Bdnf*; Fig. 2). *Il-10* was not detectable (not shown).

### Long-term effects of neonatal overfeeding on the hippocampus

**Fig. 3:**
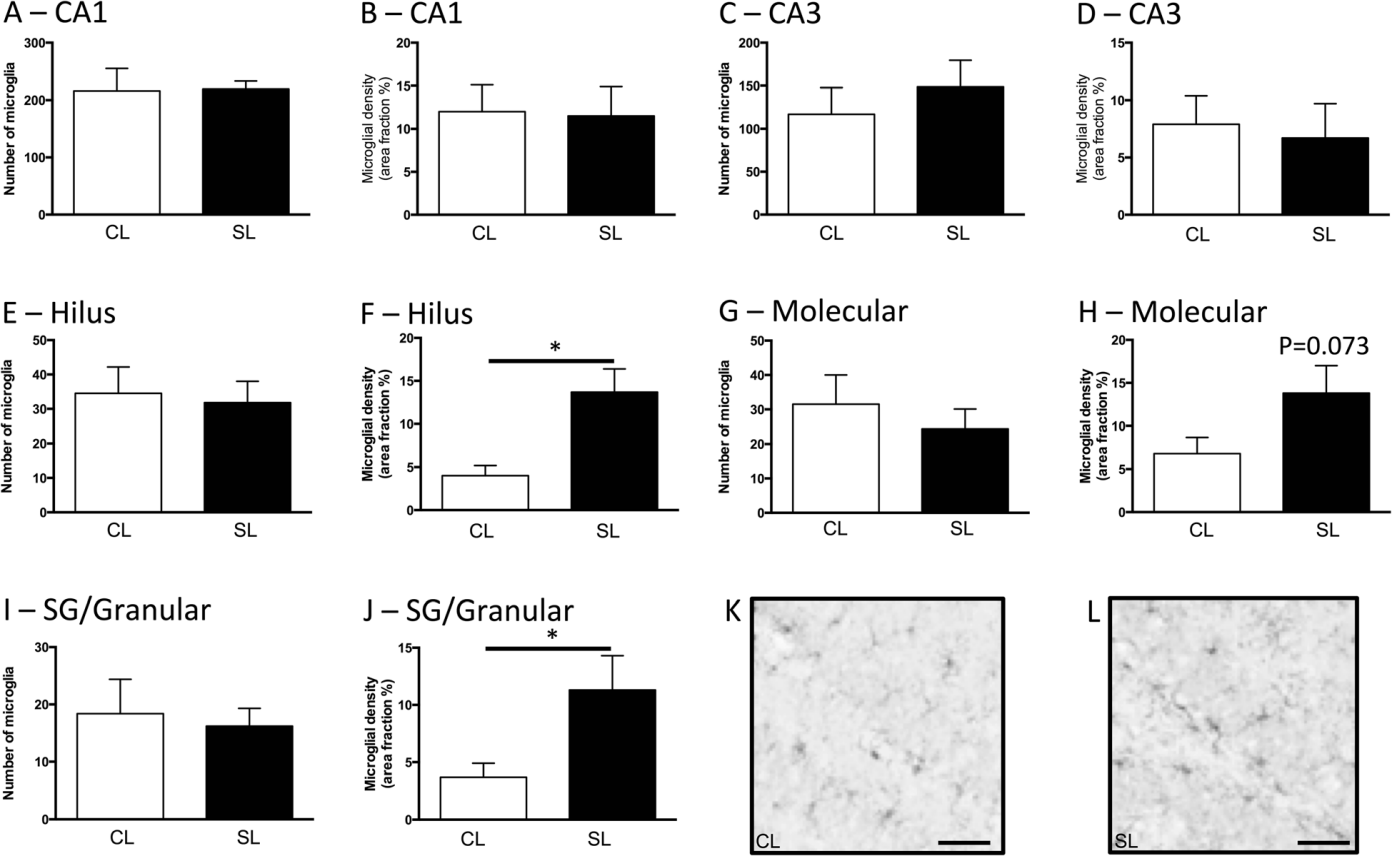
Numbers and density of ionized calcium-binding adapter molecule-1 (Iba-1)-positive cells at postnatal day 70 in rats raised in control (CL) and small (SL) litters. **a**, **b** CA1. **c**, **d** CA3. **e**, **f** Dentate gyrus hilus. **g**, **h** Molecular region. **i**, **j** Sub-granular (SG)/granular region. Data are mean + SEM. *N* = 6–12 per group. *p < 0.05. **k**, **l** Representative photomicrographs of the dentate gyrus from P70 rats illustrating differences in numbers and density of Iba-1-stained cells. *Scale bars* = 100 μm

As our previous work had shown a neonatal pro-inflammatory profile in hypothalamus with neonatal overfeeding that persisted to adulthood, we examined here if hippocampal inflammation was also maintained long term. Although there were no differences in microglial number or density in the CA1 (Fig. 3a, b) or CA3 (Fig. 3c, d), we did see an effect of early life diet on the dentate gyrus. Thus, neonatal overfeeding increased microglial density in the hilus (*t*_(10)_ = 3.65, *p* = 0.005; Fig. 3f) and sub-granular/granular (*t*_(10)_ = 2.65, *p* = 0.024; Fig. 3j) regions of the dentate gyrus compared with CL. In the molecular region, there was a *p* value between the groups of 0.073 in microglial density.

**Fig. 4:**
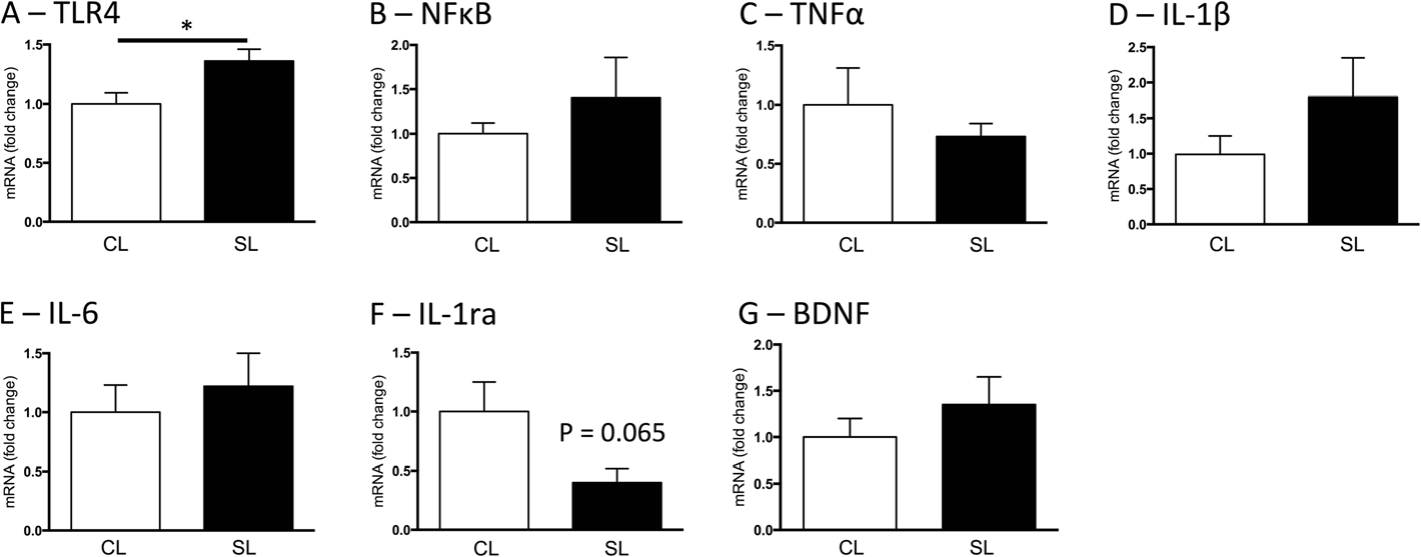
Hippocampal expression of **a** toll-like receptor 4 (*Tlr4*), **b** nuclear factor (*NF) κB*, **c** tumour necrosis factor (*TNF)α*, **d** interleukin (*IL)-1β*, **e** *IL-6*, **f** *IL-1ra*, and **g** brain-derived neurotrophic factor (*Bdnf*) at postnatal day 70 in rats raised in control (CL) and small (SL) litters. Data are mean + SEM. *N* = 6–8 per group. *p < 0.05

Neonatal overfeeding also led to an increase in hippocampal *Tlr4* compared with CL that persisted into adulthood (*t*_(13)_ = 2.68, *p* = 0.019; Fig. 4a). There were no other significant differences between the groups in hippocampal pro-inflammatory gene expression (Fig. 4) despite a trend towards a pro-inflammatory profile in SL *Il1β* (*p* = 0.189) and *Il1rn* (*p* = 0.065).

### Effects of neonatal overfeeding on cognitive function in tests of learning and memory

**Fig. 5:**
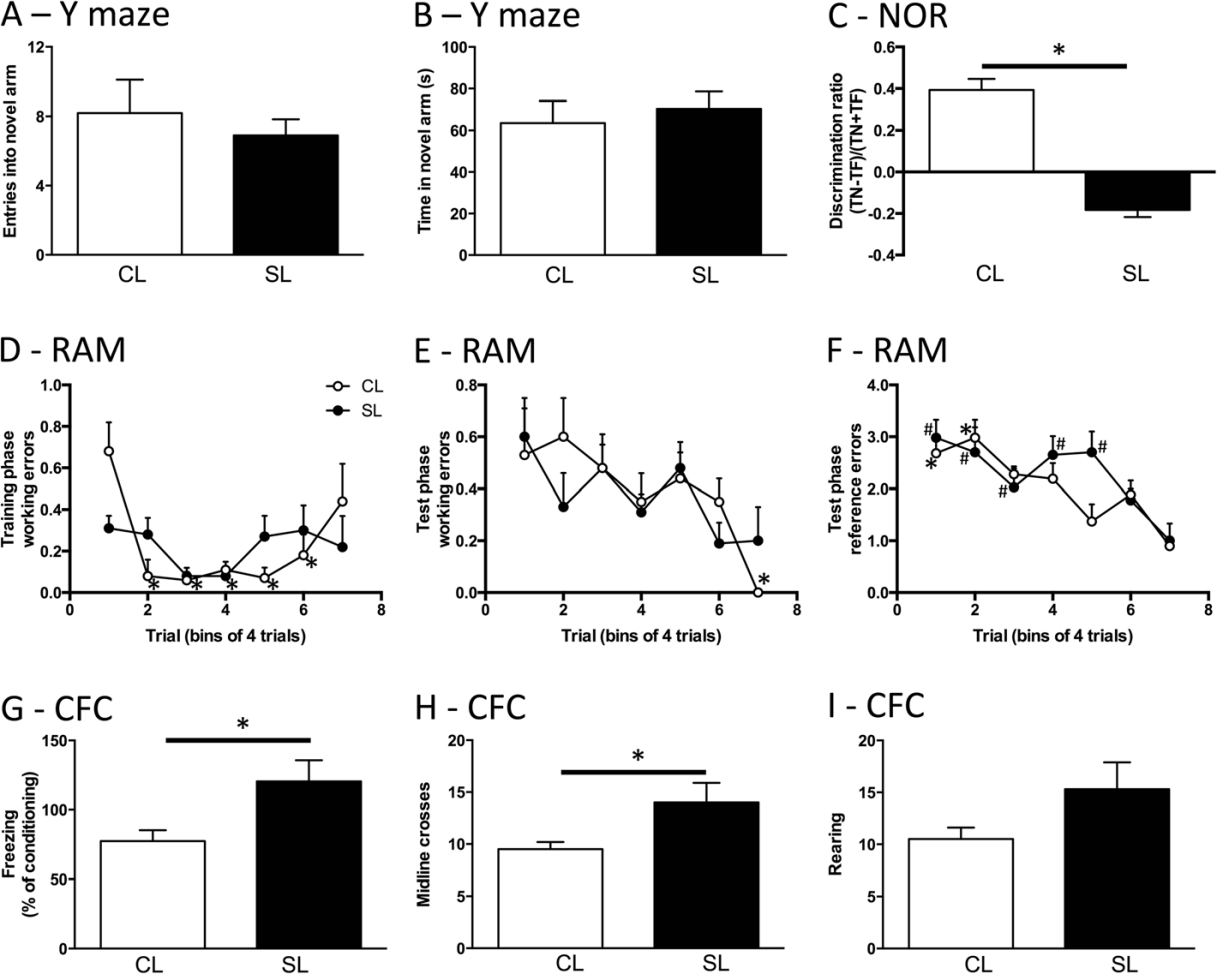
Behavioural testing of learning and memory in the Y maze (**a**, **b**), novel object recognition (**c**), radial arm maze (**d**–**f**), and contextual fear conditioning (**g**–**i**) tests in adult rats raised in control (CL) and small (SL) litters. Data are mean + SEM. **a**–**f** *N* = 10 per group. **g**–**i** *N* = 6–10 per group. **a**–**c** and **g**–**i** *p < 0.05. **d** *p < 0.05 in CL compared with CL block 1. **e**, **f** *p < 0.05 in CL compared with CL block 1 and 2; ^#^ *p* < 0.05 in SL compared with SL block 7 (criterion)

Our findings to this point indicated neonatal overfeeding conferred a mild but notable hippocampal (dentate gyrus) microgliosis, and we therefore tested if this was reflected in changes in cognitive function, specifically with respect to learning and memory. Neonatal overfeeding did not affect performance in the Y maze (Fig. 5a, b). However, subtle differences in learning and memory were seen in the novel object recognition and RAM. In the novel object recognition task, CL rats spent more time exploring the novel object (positive discrimination ratio) indicating appropriate recall of the task. SL rats, on the other hand, did not spend more time with the novel object (negative discrimination ratio), and the difference between the groups was significant (*t*_(10)_ = 9.21, *p* < 0.001; Fig. 5c).

In the RAM, the CL group commenced the training phase with a significantly greater number of working memory errors but had improved by block 2 (trials 5–8) (litter size by block interaction *F*_(6,120)_ = 2.31, *p* = 0.038; Fig. 5d). In the test phase, there was a significant improvement in working memory only in the CL group (significant effect of block *F*_(6,120)_ = 3.57, *p* = 0.003; Fig. 5e), but this was not apparent until block 7 (trial 25). The CL rats also improved significantly more quickly in their reference memory in the test phase (significant effect of block *F*_(6,120)_ = 7.91, *p* < 0.001; Fig. 5f). By block 3 (trials 9–12), CL errors were statistically not distinguishable from criterion (block 7; trial 25), whereas the SL rats were still making more errors than at criterion until block 6 (trials 21–24).

Surprisingly, the rats showed a different profile in their performance in the contextual fear conditioning test, with SL rats having better memory of the task than CL. The SL rats spent more time in freezing behaviour as a factor of training-trial freezing than CL (*t*_(14)_ = 2.78, *p* = 0.015; Fig. 5g). Neonatal overfeeding also encouraged more baseline locomotor exploration in this task (*t*_(14)_ = 2.64, *p* = 0.019; Fig. 5h).

### Effects of neonatal overfeeding on learning-induced changes in the hippocampus

**Fig. 6:**
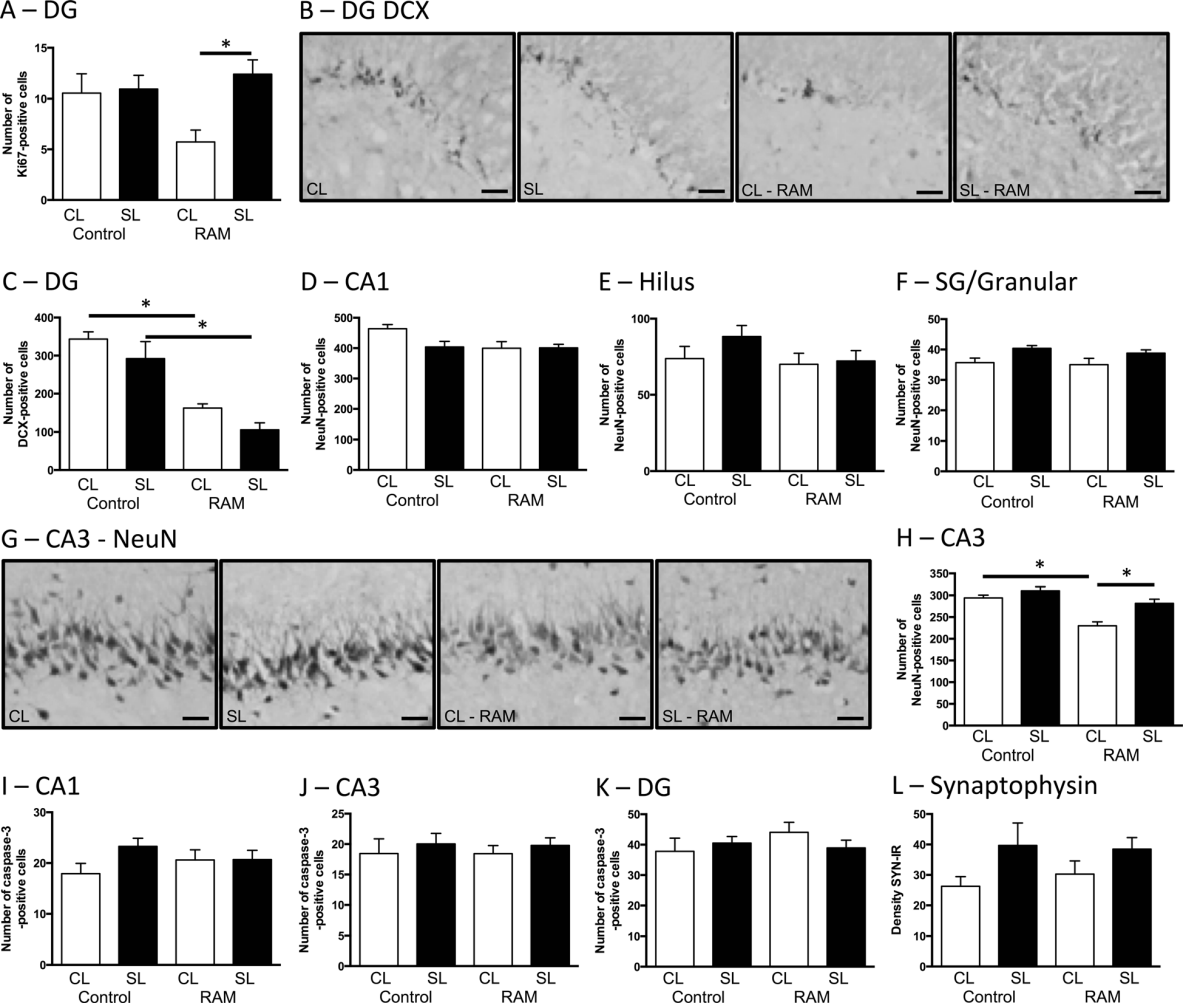
Numbers of hippocampal proliferating cells (**a**; Ki67), immature neurons (**b**, **c**; doublecortin (DCX)), total neurons (**d**–**h**; neuronal nuclei (NeuN)), apoptotic cells (**i**–**k**; caspase-3), and synaptic density (**l**; synaptophysin) at postnatal day 70 in rats raised in control (CL) and small (SL) litters under basal conditions and 24 h after the last radial arm maze (RAM) training session. Data are mean + SEM. *N* = 6–12 per group. *p < 0.05. Representative photomicrographs of hippocampal **b** DCX and **g** NeuN. *Scale bars* = 50 μm. DG dentate gyrus

Our findings with the behavioural tests led us to investigate if neonatal overfeeding leads to a change in neurogenesis or synaptogenesis in the hippocampus at 24 h after the RAM learning task in these animals in comparison to a second group of control rats that did not undergo RAM training. Contrary to our expectation that learning tasks would stimulate neurogenesis, experience of the RAM suppressed numbers of proliferating cells in CL so that following the RAM there were significantly more Ki67-positive cells in SL than CL (significant litter size by RAM interaction *F*_(1,18)_ = 4.32, *p* = 0.052; Fig. 6a). These differences are not likely to be accounted for by differences in neurogenesis as RAM also suppressed numbers of immature neurons (DCX) seen in the sub-granular/granular zone of the hippocampus, in this case in both groups (significant litter size × RAM interaction *F*_(1,26)_ = 5.32, *p* = 0.029; Fig. 6b, c). Total numbers of neurons were not affected by the learning task in the CA1 or dentate gyrus (Fig. 6d–f); in the sub-granular/granular region, there was a significant main effect of litter size with the neonatally overfed rats having more neurons overall, but there were no post hoc differences (*F*_(1,32)_ = 7.23, *p* = 0.011). In the CA3, the RAM task suppressed numbers of NeuN-positive cells in the CL so that following the RAM there were significantly more neurons in SL than CL (significant effect of litter size *F*_(1,32)_ = 14.70, *p* = 0.001 and RAM *F*_(1,32)_ = 27.50, *p* < 0.001; Fig. 6g, h). RAM did not affect apoptosis as detected by numbers of cells positive for activated caspase-3 (Fig. 6i–k), and although the neonatally overfed rats had more synaptophysin overall, there was no effect of RAM and no post hoc differences (significant effect of litter *F*_(1,17)_ = 4.85, *p* = 0.042; Fig. 6l).

**Fig. 7:**
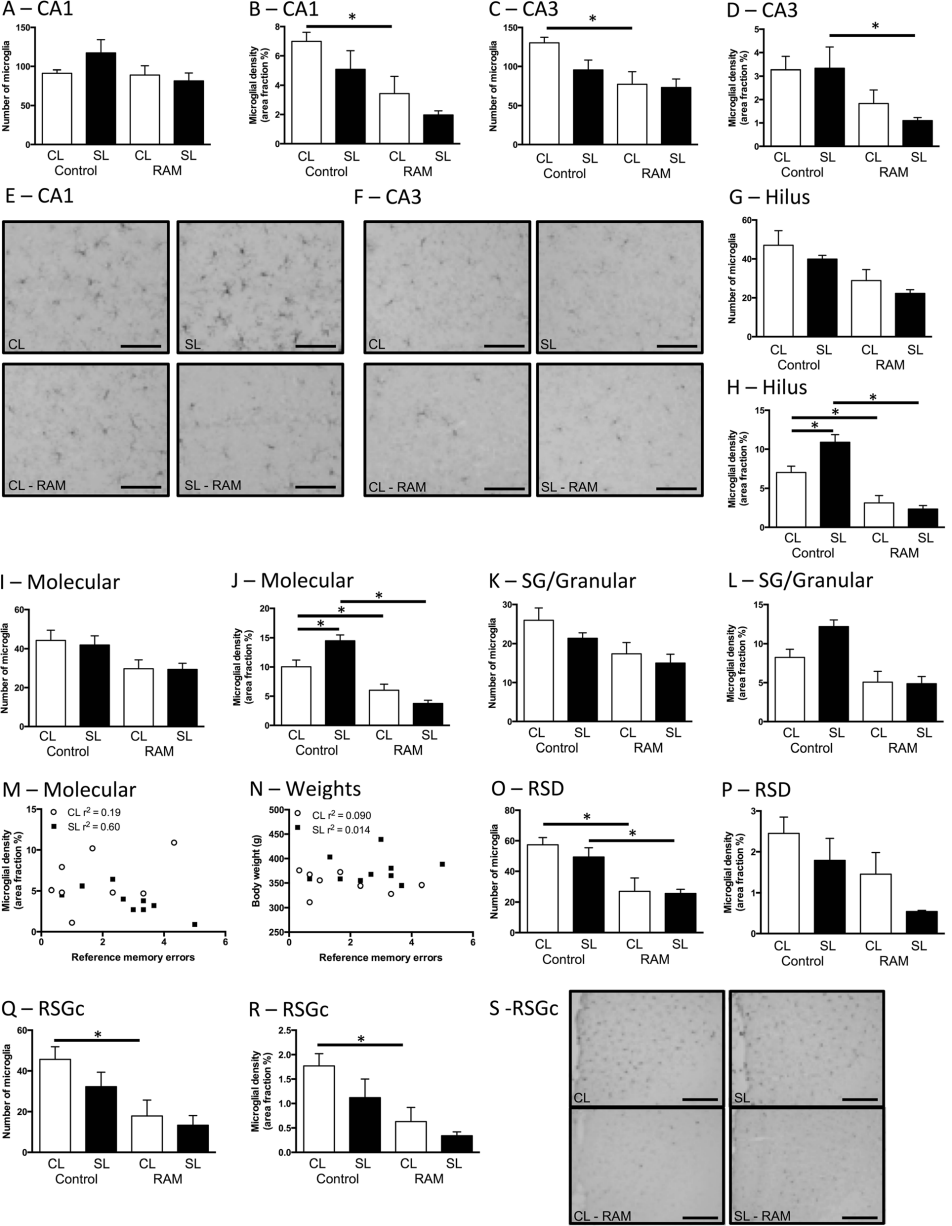
Numbers and density of ionized calcium-binding adapter molecule-1 (Iba-1)-positive cells at postnatal day 70 in rats raised in control (CL) and small (SL) litters under basal conditions and 24 h after the last radial arm maze (RAM) training session. **a**, **b**, and **e** CA1. **c**, **d**, and **f** CA3. **g**, **h** Dentate gyrus hilus. **i**, **j** Molecular region. **k**, **l** DG sub-granular (SG)/granular region. **m** Scatterplots of correlations between SL, but not CL, Iba-1 density and RAM reference errors bin 5 in the molecular region of the DG. **n** Scatterplots showing lack of correlation between CL and SL body weight and RAM reference errors bin 5. **o**, **p** Numbers and density of Iba-1-positive cells in the retrosplenial dysgranular cortex (RSD). **q**–**s** Retrosplenial granular cortex (RSGc). Data are mean + SEM. *N* = 6–12 per group. *p < 0.05. **e**, **f**, and **s** Representative photomicrographs of Iba-1. *Scale bars* = 100 μm

Previous work showing microglia’s involvement in learning and memory [16, 18] and our own findings that microglia can be programmed by neonatal overfeeding [20, 21] led us to investigate if neonatal overfeeding leads to a change in the microglial profile in the hippocampus in response to a learning task, the RAM. The experience of the RAM led to significant changes in microglial morphology, markedly suppressing microglial density throughout the hippocampus and retrosplenial cortex, particularly in CL (Fig. 7). Thus, in the CA1, the learning task suppressed microglial density in the CL group (significant effect of RAM *F*_(1,31)_ = 13.73, *p* = 0.001; Fig. 7a, b, and e). In the CA3, microglial numbers were reduced by the RAM task in CL (significant effect of RAM *F*_(1,28)_ = 9.52, *p* = 0.005; Fig. 7c, f) and density in SL (significant effect of RAM *F*_(1,27)_ = 10.98, *p* = 0.003; Fig. 7d, f). In the three regions of the dentate gyrus, the RAM reduced density in both groups and abolished basal CL and SL differences (hilus: number: significant effect of RAM *F*_(1,29)_ = 13.01, *p* = 0.001; density: significant RAM by litter size interaction *F*_(1,29)_ = 8.34, *p* = 0.007, Fig. 7g, h; molecular: number: significant effect of RAM *F*_(1,29)_ = 9.29, *p* = 0.005; density: significant RAM by litter size interaction *F*_(1,29)_ = 12.28, *p* = 0.002, Fig. 7i, j; sub-granular/granular: number: significant effect of RAM *F*_(1,29)_ = 8.28, *p* = 0.007; density: significant effect of RAM *F*_(1,29)_ = 22.65, *p* < 0.001, Fig. 7k, l). In the molecular region, we also found that the SL microglial density (but not the CL) was negatively correlated with learning (i.e. reduced microglial density was correlated with more bin 5 reference memory errors; *R*^2^ = 0.607; *p* = 0.014; Fig. 7m). There were no within-group or overall correlations for the other regions or markers and no correlation with learning and weight (Fig. 7n), despite that the SL group weighed significantly more than the CL as previously described (RAM CL = 350.1 ± 6.3 g at P57 vs SL 376.1 ± 8.9 g; *t*_(18)_ = 2.70, *p* = 0.028). The RAM reduced microglial numbers in the dysgranular region of the retrosplenial cortex in both groups (significant effect of RAM *F*_(1,32)_ = 19.59, *p* < 0.001), with a significant main effect of RAM but no group differences in density (*F*_(1,32)_ = 6.75, *p* = 0.014; Fig. 7o, p). However, numbers and density were only affected in CL in the granular region (number: significant effect of RAM *F*_(1,31)_ = 12.14, *p* = 0.001; density: significant effect of RAM *F*_(1,30)_ = 12.91, *p* = 0.001; Fig. 7q–s), the learning task thus having clear effects on microglial profiles in these regions, particularly in CL rats.

As our findings with the fear conditioning experiment suggested the amygdala responses may be independent of the hippocampal ones, we also extended our analysis to amygdala. We found a significant effect of RAM on numbers and density of microglia in the central amygdala (number: *F*_(1,31)_ = 5.09, *p* = 0.031; density: *F*_(1,31)_ = 7.31, *p* = 0.011), on numbers in the basolateral amygdala (*F*_(1,31)_ = 4.28, *p* = 0.047), and on density in the medial amygdala (*F*_(1,31)_ = 5.79, *p* = 0.022), with RAM experience suppressing both number and density. However, there were no significant group differences with post hoc tests (Additional file 1: Figure S1).

## Discussion

This study is the first to (A) show the effects of postnatal overfeeding on hippocampal inflammation in association with cognition; (B) determine whether the cognitive effects of diet can persist long term if the overfeeding takes place (for only 3 weeks) in the neonatal period, in contrast with the adult where dietary effects are somewhat reversible; and (C) examine the changes that occur in microglia in cognitive-processing regions in response to a learning task (either in the overfed or control state).

Diet in the early life period is important for programming many long-term aspects of physiology, including priming hypothalamic microglia to be more reactive to a neuroimmune challenge [20]. In this study, we show that early life overfeeding can also stimulate acute and long-term changes in the pro-inflammatory (*Tlr4*) and microglial profile in the hippocampus, and this is associated with reduced microglial sensitivity to a spatial memory task and poorer performance in the task. Here we have shown that early life overfeeding leads to increases in the number and density of cells expressing Iba-1 in the CA1 region of the hippocampus in early postnatal life (P14). While Iba-1 is also expressed in non-microglial CNS macrophages, we are confident that our analysis is principally of microglia since these exist mainly within the parenchyma [43], whereas non-microglial macrophages are mostly considered to be a hematogenous cell type and thus localize primarily around blood vessels of the brain, in the choroid plexus, perivascular space, and meninges [43], regions that were not included in our analysis. Nonetheless, we cannot rule out that our assessed population does not include other macrophages. These differences in Iba-1-expressing cells in the CA1 normalize by the time the rats reach adulthood, but in neonatally overfed adults we see increased density in three sub-regions of the dentate gyrus and increased *Tlr4* expression in the hippocampus as a whole. While it is noteworthy that we see significant differences in *Nfκb* and *Tnfα* in neonates and in *Tlr4* in the adults in whole hippocampus, it will be useful to examine regional differences in cytokine gene expression in the future. To determine if these changes in Iba-1 density were associated with differences in cognitive function, we tested the rats in several learning and memory tests. Neonatally overfed rats performed less successfully in tests of hippocampal-dependent working and spatial memory. They did not recognize a previously encountered object in the novel object recognition test and were less efficient in their memory acquisition in the RAM, taking longer to approach the learning criterion. Collectively, our data suggest that despite indications of microglial priming in the dentate gyrus prior to RAM, the neonatally overfed rats’ hippocampus and retrosplenial cortex are actually less sensitive to the effects of a learning challenge than control rats are.

Spatial learning is known to specifically modulate neurogenesis and apoptosis to promote the survival of relatively mature developing neurons and induce the death of less mature ones [44, 45]. Presumably, this is to preserve those neurons that are already capable of receiving depolarizing GABAergic inputs and communicating glutamatergic outputs at the expense of those that are not and reduce noise in the network [45]. Indeed, an attenuation of neurogenesis in the late, consolidation, phase of learning is essential for accurate memory performance, and rats with the highest learning-induced decrease in BrdU-labelled cells perform the best in the Morris Water Maze test for spatial memory [44]. Prevention of apoptosis in this phase, with caspase-3 inhibition, delays acquisition of spatial memory in this task, reflecting impaired learning [45]. Although both groups of our rats had similar levels of newborn neurons, as detected with DCX, only the CL had reduced cell proliferation and reduced total neuron numbers in the CA3, potentially indicating only the CL was capable of stimulating apoptosis of very young neurons early on during learning and suppressing cell proliferation until the task had been mastered. Given the well-known role of glucocorticoids in neurogenesis [46], it is possible that different corticosterone levels under basal conditions or in response to the RAM between the groups could account for some of our effects. We did not measure corticosterone concentrations in this study, but we have previously shown that there are no significant differences between basal corticosterone in male or female neonatally overfed adult rats compared with controls [24, 27, 47]. We have also seen that males have no differences in their corticosterone responses to restraint stress [27] and no differences in hypothalamic or hippocampal glucocorticoid or mineralocorticoid receptor expression under basal conditions [47]. These findings make it unlikely that the corticosterone response to RAM is substantially different between the groups. However, we have seen that neonatally overfed males have a slower corticosterone response to LPS [24, 47], so this remains a possibility. It is noteworthy that our RAM task was terminated immediately upon our rats reaching criterion, after 14 days (25 sessions) of training. This timing therefore does not necessarily correspond to that of the studies conducted with the Morris Water Maze where learning acquisition had stabilized [44, 45], which may be why we do not see increases in apoptosis in either group. Additionally, apoptosis may be occurring via caspase-3-independent pathways at the late phase in our rats.

In addition to an inability to suppress cell proliferation and CA3 neuron numbers, microglia were not affected by the RAM to the same degree in the neonatally overfed rats. This finding was contrary to our initial hypothesis that early life overfeeding would leave hippocampal microglia primed to hyper-respond to a challenge, resulting in a pro-inflammatory profile after the learning task [11]. Microglia are known to play a key role in learning and memory, regulating apoptosis and synaptic plasticity [48]. High-fat diet in adulthood can reversibly increase microglia’s internalization of synaptic contacts leading to memory deficits [23]. Short-term microglial depletion has also been shown to affect synaptic remodelling, reduce spontaneous glutamate release in the motor cortex, and impair memory [49]. On the other hand, longer-term microglial ablation in a different model can significantly improve memory performance [50]. In this latter case, mice given PXL3397, an inhibitor of colony-stimulating factor 1 expressed on microglia, for 2 months had a 99 % depletion of microglia across the brain. These mice were significantly better than control mice at remembering the location of the escape route in the Barnes maze test with no differences in locomotion or other behaviours and no differences in contextual fear conditioning. This memory enhancement in the Barnes maze was not apparent after only 3 weeks microglial depletion [50]. This study and our own findings that the physiological response of the normal (control) rat to the RAM is to reduce numbers of microglia in the hippocampus and retrosplenial cortex suggest that the ability to reduce the microglial input at certain stages of the task may be crucial to successful learning. It appears from our data that the neonatally overfed rats are less efficient at doing this. However, our data also suggest that a certain threshold of microglial activation is also necessary for appropriate learning, since in the molecular region of the dentate gyrus, microglial density was negatively correlated with learning performance in the neonatally overfed rats.

In contrast to our novel object recognition and RAM results, the findings from the contextual fear conditioning test suggest that neonatally overfed rats actually have enhanced memory. This appears counterintuitive if neonatal obesity causes cognitive dysfunction and contrast's with adult models where high-fat diet (12 and 20 weeks) causes impairments in contextual fear conditioning [22]. However, this finding has an interesting precedent in the literature. Boitard and colleagues have recently shown that adolescent, but not adult, high-fat diet enhances amygdala-dependent, i.e. emotional, memory [51]. In this work, they determined rats fed with high-fat diet in adolescence are more effective at remembering an odour associated with illness (LiCl-paired odour) and an auditory tone associated with footshock than control fed rats (and high-fat diet-fed adults) [51]. Their work suggests amygdala-dependent memory is enhanced in obese adolescent rats, and this elevated fear response is related to hypothalamic-pituitary-adrenal (HPA) axis dysfunction, since a glucocorticoid receptor antagonist prevents the obesity-associated emotional memory and basolateral amygdala synaptic plasticity. Interestingly, we have seen that neonatal overfeeding also alters HPA axis function long term, with neonatally overfed rats having exacerbated HPA axis responses to an immune challenge (males and females) [21, 24] and a psychological stress (females only) [27]. Together, our findings suggest that early life overfeeding may specifically and differentially influence both hippocampal and amygdala-based learning and memory, making the animal more responsive to amygdala-dependent aversive learning, but less efficient at hippocampal-dependent non-aversive memory tasks. This idea is somewhat reflected in our data showing an effect of RAM to suppress microglial numbers and density in the amygdala, an effect that seems to be primarily carried by the SL group. There were no relevant post hoc differences here, however, and it will be interesting to test amygdala microglial responses to fear conditioning tasks. There also remains the possibility that the responses we see in the RAM and contextual fear conditioning tests are different because the former is a learning task acquired over multiple days requiring constant updating of newly acquired information via neuronal plasticity, while the latter is a single-trial learning task with recall tested, in this case, only after 24 h. This possibility remains to be tested, but we also saw deficits in our neonatally overfed rats in their ability to recall objects in the novel object recognition task.

Our findings that there were no within-group or overall correlations between learning and weight suggest that the long-term effects of early life diet on the brain are more important than weight per se for cognitive function. Mechanistically, it remains to be determined how might early life overfeeding lead to long-term changes in microglial responsiveness. In adults, a diet high in fat, particularly saturated fat [52], can lead to a systemic pro-inflammatory profile [11, 53–55] with a resulting increase in circulating pro-inflammatory cytokines that contribute to systemic insulin resistance and also stimulate an inflammatory response at the level of the hypothalamus [11, 53–55]. The hypothalamic response to fat is remarkable, with microgliosis in this region occurring in rodent models as early as 24 h after they commence a high-fat diet [19], and is linked with the death of pro-opiomelanocortin cells after 2 weeks [19]. While high-fat diet clearly and quickly contributes to hypothalamic inflammation, and this process significantly disrupts feeding and metabolism, how central inflammation might influence cognition may be less direct. It is likely that high-fat diet-induced microgliosis results in synaptic remodelling, neuronal apoptosis, and impaired neurogenesis in the hypothalamus, also disrupting hypothalamic connectivity with brain regions important in cognitive function, such as the hippocampus and retrosplenial cortex. Prolonged high-fat diet may also directly contribute to inflammation in these extra-hypothalamic regions. As such, hippocampal IL-1β is elevated after 12 and 20 week high-fat diet and the associated cognitive dysfunction improved by central IL-1RA [22]. Together these mechanisms are likely to lead to ongoing deficits in cell signalling and connectivity, with impairment in cognitive function (discussed in [11]). In neonates, a similar process may occur, but because the microglia are maturing at this time the changes may be more permanent. Walker’s group has demonstrated a lasting role for leptin in hippocampal development, so it may be that elevated leptin, occurring with neonatal overfeeding, alters neurogenesis and synaptogenesis in the early phase, permanently changing the way this region responds to a memory task [56, 57]. In terms of microglia, Bilbo and colleagues have shown that early life perturbations can lead to microglial priming such that they retain a more activated phenotype throughout life and are more sensitive to stimuli. For instance, neonatal infection with *Escherichia coli* at P4 can prime microglia to be more reactive to an LPS challenge throughout life [30]. These neonatally immune-challenged rats also have impaired contextual memory in the presence of a novel neuroimmune challenge [58], but the microglial response to the memory task was not assessed in this study. In this context, we speculate neonatal overfeeding is also priming hippocampal microglia to be sensitive to neuro-inflammatory stimuli but correspondingly less able to down-regulate or reduce their input when needed for a learning task, and thus, their role in regulating neurogenesis and synaptic plasticity is impaired.

## Conclusions

In summary, we have shown that neonatal overfeeding leads to long-term cognitive deficits and this is associated with reduced ability to regulate neurons and microglia in the context of a learning task. We suggest that microglial priming may be involved. These data may partially explain why some individuals with obesity display cognitive dysfunction and some do not, i.e. the early life dietary environment may have a vital long-term contribution.

## Ethics approval

We conducted all procedures in accordance with the National Health and Medical Research Council Australia Code of Practice for the Care of Experimental Animals and the RMIT University Animal Ethics Committee approval.

### Consent for publication

Not applicable.

### Availability of data and materials

The datasets supporting the conclusions of this article are available upon request.

## Additional file

Additional file 1: Figure S1. Numbers and density of ionized calcium-binding adapter molecule-1 (Iba-1)-positive cells at postnatal day 70 in rats raised in control (CL) and small (SL) litters under basal conditions and 24 h after the last radial arm maze (RAM) training session. A, B) Central amygdala (CeA). C, D) Medial amygdala (MeA). E, F) Basolateral amygdala (BLA). Data are mean + SEM. *N* = 6-12 per group. (TIFF 2231 kb)

## Abbreviations

ANOVA: analyses of variance
*Bdnf*: brain derived neurotrophic factor
BLA: basolateral amygdala
CeA: central amygdala
CL: control litter
DAB: diaminobenzidine
DCX: doublecortin
DG: dentate gyrus
HPA: hypothalamic-pituitary-adrenal
HRP: horseradish peroxidase
Iba-1: ionized calcium-binding adapter molecule-1
IL: interleukin
ITI: inter-trial interval
MeA: medial amygdala
*Nfκb*: nuclear factor κB
NeuN: neuronal nuclei
P: postnatal day
PBS: phosphate-buffered saline
PVN: paraventricular nucleus of the hypothalamus
qrt-PCR: real-time reverse transcriptase polymerase chain reaction
RAM: radial arm maze
ROI: region of interest
RSD: retrosplenial dysgranular cortex
RSGc: retrosplenial granular cortex
