# Supplementary figures and images for: Early life overfeeding impairs spatial memory performance by reducing microglial sensitivity to learning
Source: J Neuroinflammation. 2016 May 18;13:112. doi: 10.1186/s12974-016-0578-7 (PMC4872342; doi:10.1186/s12974-016-0578-7)

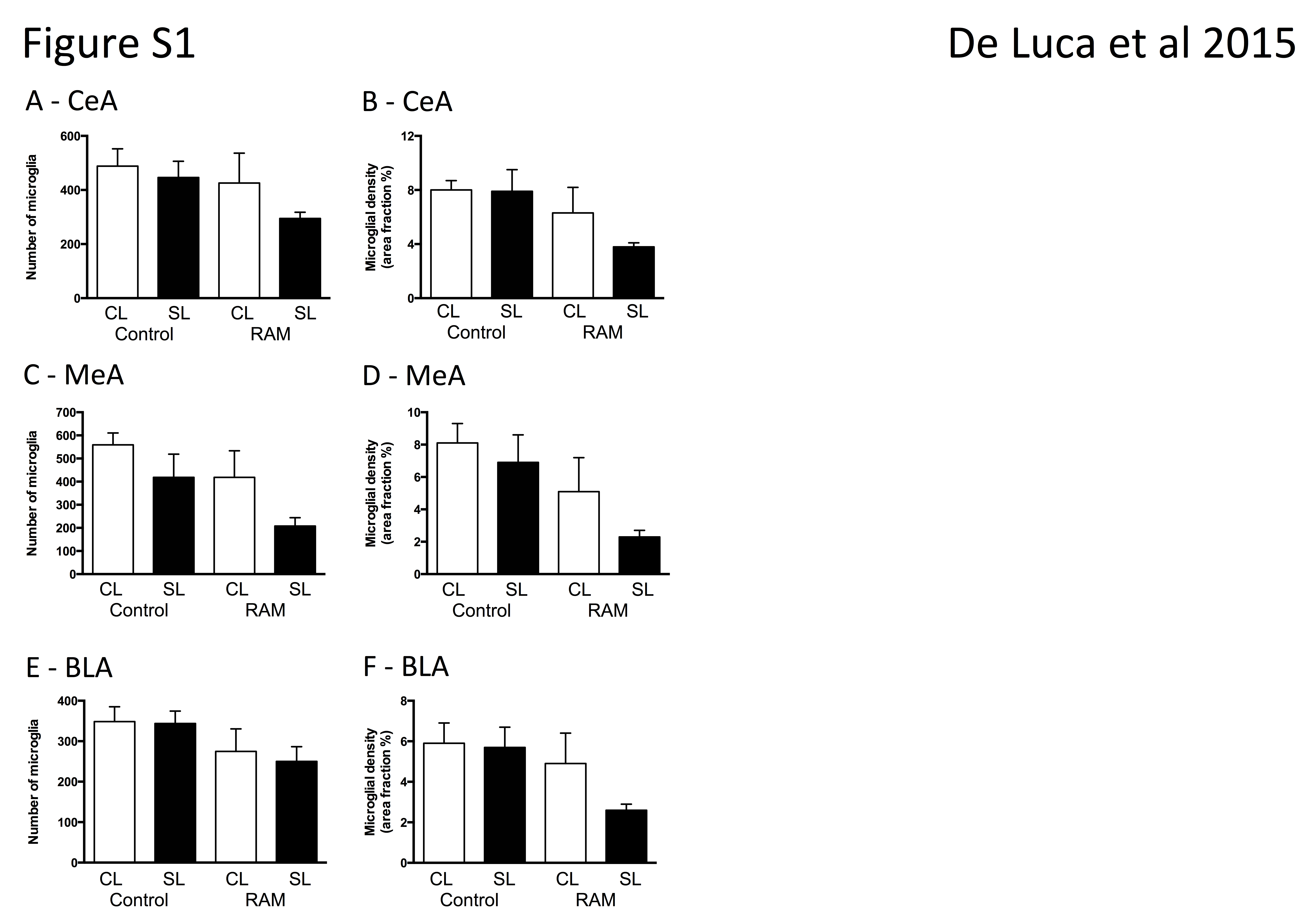

Supplement: Additional file 1: Figure S1. — Numbers and density of ionized calcium-binding adapter molecule-1 (Iba-1)-positive cells at postnatal day 70 in rats raised in control (CL) and small (SL) litters under basal conditions and 24 h after the last radial arm maze (RAM) training session. A, B) Central amygdala (CeA). C, D) Medial amygdala (MeA). E, F) Basolateral amygdala (BLA). Data are mean + SEM. N = 6-12 per group. (TIFF 2231 kb) [file 12974_2016_578_MOESM1_ESM.tiff]
